# Feeding Frequency Modulates the Intestinal Transcriptome Without Affecting the Gut Microbiota in Pigs With the Same Daily Feed Intake

**DOI:** 10.3389/fnut.2021.743343

**Published:** 2021-10-29

**Authors:** He Zhang, Pengke Xia, Lufen Feng, Menglan Jia, Yong Su

**Affiliations:** ^1^Laboratory of Gastrointestinal Microbiology, Jiangsu Key Laboratory of Gastrointestinal Nutrition and Animal Health, College of Animal Science and Technology, Nanjing Agricultural University, Nanjing, China; ^2^National Center for International Research on Animal Gut Nutrition, Nanjing Agricultural University, Nanjing, China

**Keywords:** different feeding frequencies, intestinal microbiota, transcriptomic profiling, growing pig, ileum, colon

## Abstract

The objective of this study was to elucidate the impacts of irregular eating patterns on gut microbiota and transcriptomic responses in a pig model with different feeding regimens. The experiment involved 24 growing pigs (Duroc × Landrace × Large White, 48 days of age) which were randomly allocated to one of three feeding patterns: one-meal (M1), three-meals (M3), or five-meals (M5) per day with the same daily feed intake. The results showed that different feeding frequencies had no significant effects on the microbial composition of ileal digesta, colonic digesta, colon mucosa, as well as the concentration of SCFAs in colonic digesta. Mucosa transcriptomic profiling data showed the pathways related to vitamin metabolism were enriched in the ileum and colon of pigs in the pairwise comparison between M3 and M1 groups. On the other hand, the pathways related to lipid metabolism were enriched in the ileum and colon of pigs in the pairwise comparison between M5 and M1 groups. Lastly, the pathways related to protein metabolism were enriched in the colon in the pairwise comparison between M3 and M1 groups, M5 and M1 groups, M5 and M3 groups, while the ileum was not enriched. Differentially expressed genes (DEG) related to metabolism showed that carbohydrate transport was suppressed in the ileum and enhanced in the colon in M5 and M3 groups compared with the M1 group. Compared with the M3 group, carbohydrate transport in the ileum was enhanced in the M5 group, while in the colon was inhibited. With the increase of feeding frequency, the catabolism, biosynthesis, and transport of lipid in the ileum were suppressed, while those in the colon were enhanced. Compared with the M1 group, amino acid transport in the ileum and colon in the M3 group was enhanced. Amino acid catabolism in the ileum in the M5 group was enhanced compared with M1 and M3 groups. In summary, different feeding frequencies affected the transport of carbohydrate, lipid, and amino acid in the ileum and colon, and affected the catabolism and biosynthesis of lipid in the ileum and colon with a low impact on intestinal microbiota.

## Introduction

With the pace of life accelerated, eating habits become increasingly diverse, such as skipping breakfast and/or late-night eating, increasing the risk of obesity and metabolic diseases, which have attracted the attention of those in the public health field, and effective medicine or approaches are needed to prevent and treat (1, 2). As a part of the strategies to reduce energy intake (diets, drugs, and bariatric surgery) (3) and to increase energy output (exercise and non-exercise movement) (4), meal timing and frequency have an important impact on weight control and weight loss (5, 6). Researchers are looking for a simple solution to weight loss and decrease their metabolic diseases, and they have recently increased in popularity of low overall feed intake, however, research on the effects of irregular eating patterns on weight control and metabolism under the condition of an equal amount of feed intake is scarce. Our previous study found different feeding frequencies significantly affected the growth performance of growing pigs with the same feed intake, indicating that meal frequency as a strategy had an important impact on weight control (7).

In recent years, more and more evidence has indicated that diet composition (8), nutritional concentrations (9), and eating habits (10) could shape microbial communities. Researchers believe that diet and eating habits represent the main contributors to obesity-related alterations in the intestinal microbiota due to they can shape the environment of intestinal bacteria. Emerging studies suggest that the presence of diurnal oscillations in the intestine microbiota, and mealtime is now as important as dietary composition in entraining microbiome rhythmicity (11–13). A stable functional microbiota contributes to host health, including nutritional, metabolic, and immune homeostasis, and metabolic function. In previous studies, we confirmed that the apparent total tract digestibility of crude protein was significantly higher in three-meal daily and five-meal daily groups than that in one-meal groups (7). In addition, the activities of digestive enzymes, including trypsin, chymotrypsin, lipase, and maltase were affected by different feeding frequencies. However, the underlying mechanism of feeding frequency affecting nutrient digestion and absorption and the possible role of intestine microbiota is unknown.

Le Naou et al. (14) characterized the effects of feeding frequency on postprandial metabolism and circulation nutrients, however, there is still a paucity of information regarding the possible alterations on intestinal microbiota composition and intestinal mucosa transcriptome profile. The present study hypothesized that different feeding regimens could change the gene expression profile of intestinal mucosa transcriptome and affect the composition of intestinal microbiota. Because it is similar in anatomy and metabolism to humans, the pig has been recognized as an ideal model for human nutrition research (15). Therefore, the growing pig with different feeding regimens in the study was used to investigate the effects of feeding frequency on intestinal microbial composition, microbial metabolites, and intestinal mucosa transcriptional level under the same daily feed intake.

## Materials and Methods

### Experimental Animals, Design, and Diet

A total of 24 crossbred (Duroc × Landrace × Yorkshire, initial body weight = 15.64 ± 0.69 kg, 48 days of age) growing barrows from a commercial farm (Jiangsu, China) were randomly allocated to the one-meal daily feeding (M1) group, three-meal daily feeding (M3) group, and five-meal daily feeding (M5) group, with each group consisting of eight replicates (pens) and with one pig per pen. Firstly, pigs in the M1 group were fed once at 7:00 a.m. of each experimental day according to the standard feeding requirement of the National Research Council (NRC, 2012) (16). Secondly, pigs in the M3 group were fed one-third of the standard feeding requirement at 7:00 a.m., 12:00 noon, and 5:00 p.m., respectively. Lastly, pigs in the M5 group were fed one-fifth of the standard feeding requirement at 7:00 a.m., 9:30 a.m., 12:00 noon, 2:30 p.m., and 5:00 p.m., respectively.

All pigs were housed individually in metal floor cages (0.85 m height × 1.2 m length × 0.7 m width) with a smooth-walled pan and a feeder. The pigs had free access to water *via* a nipple drinker during the 1-month trial period. The temperature of the pig house was maintained at 25 ± 2°C. All pigs were kept at a 24-h light-dark cycle, with lights being turned on from 7:00 a.m. to 7:00 p.m. To examine the effect of feeding frequency on intake behavior, feed intake was recorded every two-and-a-half hours from 9:30 a.m. to 7:30 p.m. for three consecutive days from the 16th day of the feeding experiment. The composition and nutrient levels of the diet are shown in Supplementary Table 1.

### Tissue Sample Collection

The feeding experiment lasted for 30 d, and the pigs were slaughtered on d 31 after an overnight fast. All pigs were anesthetized using an intravenous injection of 4% sodium pentobarbital solution (40 mg/kg BW) from 8:00 a.m. to 10:00 a.m. The anterior ileum luminal digesta and proximal colonic luminal digesta were collected and stored at −28°C for further microbial structure analysis. The anterior ileum and colon tissues were washed with sterile phosphate-buffered saline (PBS) (pH 7). The anterior ileum mucosal samples were collected by scraping the luminal surface with a sterile glass slide and stored in liquid nitrogen immediately and then transferred to a −80°C low-temperature refrigerator (Haier, China) for further transcriptome sequencing analysis. The colon mucosal samples were collected by scraping the luminal surface with a sterile glass slide and stored in liquid nitrogen immediately and then transferred to a −80°C low-temperature refrigerator for further microbial structure and transcriptome sequencing.

### DNA Extraction, MiSeq Sequencing, and Data Processing

The total genomic DNA of bacteria in the anterior ileal digesta, colonic digesta, and colon mucosa was extracted using a commercially available stool DNA extraction kit (QIAamp DNA Stool Mini Kit, Qiagen, Hilden, Germany) according to the instructions of the manufacturer. The bead-beating was used before extraction to ensure that the cell walls of bacteria were broken. The V3-V4 regions of the bacterial 16S rRNA gene were amplified using universal primers (341F 5′-CCTAYGGGRBGCASCAG-3′ and 806R 5′-GGACTACNNGGGTATCTAAT-3′) following methods as previously described (17). PCR products were purified by the AxyPrep DNA Gel Extraction Kit (Axygen Biosciences, Union City, CA, USA). Purified amplicons were pooled in paired-end sequences (2 × 250) on an Illumina MiSeq platform (Biozeron, Shanghai, China) according to standard protocols. Operational taxonomic units (OTUs) were clustered with a 97% similarity cutoff using UPARSE (version 7.1; http://drive5.com/uparse/, California, USA) and chimeric sequences were identified and removed using UCHIME. The raw reads of MiSeq sequencing of 16S ribosomal RNA (rRNA) gene were deposited into the GenBank Sequence Read Archive database under accession number PRJNA748604, PRJNA748609, and PRJNA748605. Species richness indices (Chao 1) and diversity indices (Shannon) were calculated using the MOTHUR (version 1.36.1, Michigan, USA). UniFrac-based principal coordinates analysis (PCoA) was conducted to assess the structural similarity between communities. The relative abundance of dominant bacteria (abundance > 1%) at the genus and OTU levels in the ileal digesta, colonic digesta, and colon mucosa were analyzed.

### RNA Preparation and Sequencing

Total RNA was extracted from the anterior ileum mucosal and colon mucosal of pigs using the RNeasy mini kit (Qiagen, Hilden, North Rhine-Westphalia, Germany) according to the instructions of the manufacturer. The RNA integrity number (RIN) value was determined using the Agilent 4200 Bioanalyzer (California, USA) and high sensitivity RNA screen tape kit according to the instructions of the manufacturer. Samples with RIN values >8.5 were selected for dilution. The cDNA library was constructed using a NEBNext® UltraTM II Directional RNA Library Prep Kit for Illumina® and was successfully sequenced on an Illumina HiSeq X10 (Illumina, USA) sequencer with a pattern of PE150. The RNA-seq datasets were deposited into the GenBank Sequence Read Archive database under accession numbers PRJNA747849 and PRJNA747852.

### Analyses of RNA-Seq Data

The adaptor sequences and low-quality sequence reads (containing adapter reads, poly-N reads, and reads with low base recognition rate) were removed from the raw data sets, and then clean data sets were obtained. The clean reads were mapped to reference sequences and/or reference genomes. The clean reads were mapped to the reference pig genome (Sus scrofa 11.1). Gene expression levels were quantified by fragments per kilobase of transcript per million fragments (FPKM) methods (18). Differentially expressed genes (DEGs) of M3 vs. M1, M5 vs. M1, and M5 vs. M3 were performed using the DESeq2 R package (version 1.16.1, NorthCarolina, USA) (19). *P* < 0.05 and fold change (FC) ≥ 1.5 or <0.67 were set as DEG threshold. The Gene Ontology (GO) and Kyoto Encyclopedia of Genes and Genomes (KEGG) enrichment analysis of the differential genes were performed by clusterProfiler (version 3.16, Guangdong, China) and org.Ss.eg.db (version 3.11.1, Washington, USA) R-package. The statistical analyses of the KEGG enrichment were set as *P* < 0.05.

### Microbial Metabolite Analysis

Concentrations of short-chain fatty acids (SCFAs) were determined by gas chromatography according to the method described in a previous study (20).

### Validating the Expression of DEGs by RT-qPCR

To validate the veracity and reliability of the transcriptome data, 12 DEGs were randomly selected for conducting RT-qPCR validation. The RT-qPCR of the target genes was performed using the ABI 7300 real-time PCR system (SDS, Foster City, CA, USA) with fluorescence detection of SYBR Green PCR Kit (TaKaRa, Co. Ltd. Dalian, China). All primers were synthesized by Invitrogen Life Technologies (Invitrogen, Shanghai, China) with the sequences are shown in Supplementary Table 2. The relative quantification of the gene expression differences was calculated with the formula 2^−ΔΔCt^ (21).

### Statistical Analysis

All data were analyzed using SPSS version 22 software (SPSS Inc., Chicago, IL, USA) as a randomized complete block design. Differences in the microbial abundance (at phylum, genus, and OTU levels) were analyzed by the Mann-Whitney *U*-test, and data were expressed as medians. The data of bacterial metabolites, diversity indices, richness indices, and RT-qPCR were tested using one-way ANOVA, followed by Duncan's test, and the data are presented as the mean ± SEM. Significant differences were considered when *P* < 0.05.

## Results

All the pigs were kept healthy during the experiment. No difference in the average daily feed intake was found among the three groups. The final body weight and average daily gain of pigs in the M3 and M5 groups were significantly higher than that in the M1 group, and the feed, wherein the gain was lower in M3 and M5 groups than that in the M1 group. Moreover, there were no significant differences in final body weight, average daily gain, and feed, specifically the gain between M3 and M5 groups (7). The eating patterns were significantly different among the three groups. Pigs in the M1 group consumed a greater proportion (~79%) of feed during the morning, pigs in the M3 group had the same feed intake in the three feeding periods (7:00 a.m.−9:30 a.m., 12:00 p.m.−2:30 p.m., and 5:00 p.m.−7:30 p.m.), and pigs in the M5 group had the same feeding fate at each period during the day.

### Ileal Digesta, Colonic Digesta, and Colon Mucosa Microbial Community

**Figure 1:**
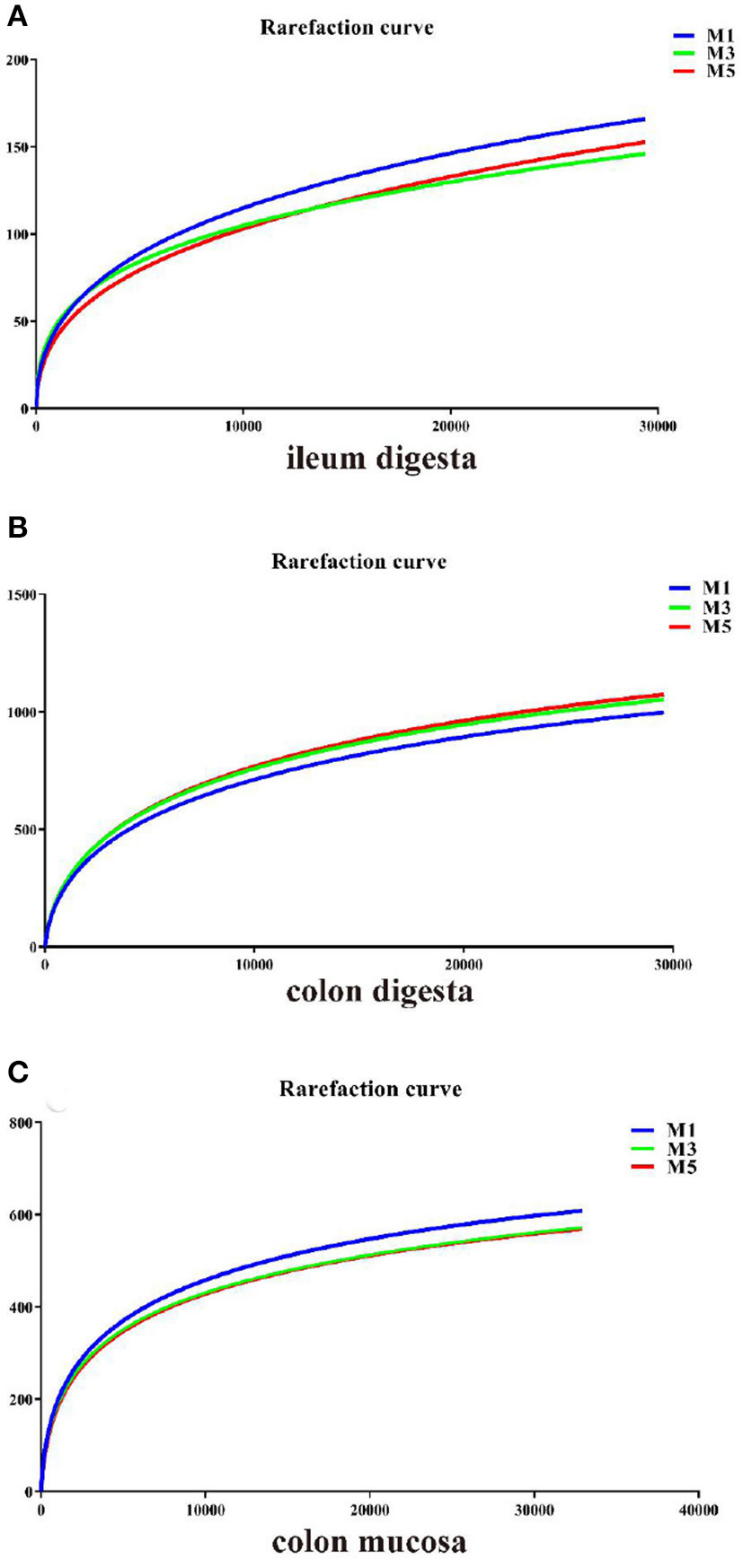
Rarefaction curves comparing the number of sequences with the number of operational taxonomic units (OTUs) found in the 16S rRNA gene libraries from microbiota in the ileal digesta, colonic digesta, and colon mucosa of pigs in three groups. M1, mean of eight repetitions in pigs fed one meal daily; M3, mean of eight repetitions in pigs fed three meals daily; M5, mean of eight repetitions in pigs fed five meals daily.

**Figure 2:**
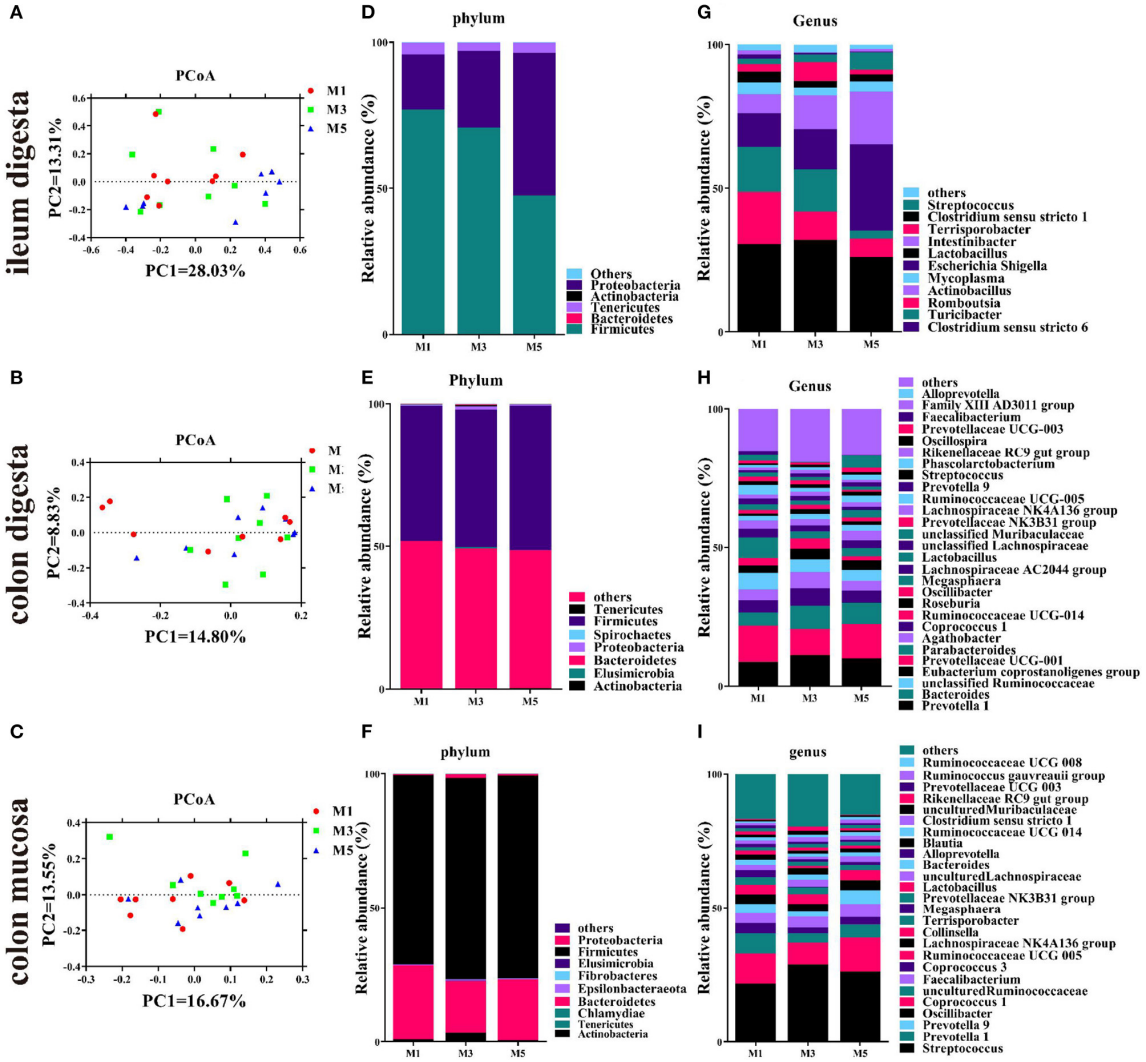
Effect of feeding frequency on intestinal microbiota of pigs. Unweighted unifrac principal coordinates analysis (PCoA) plot of ileal digesta microbiota **(A)**, colonic digesta microbiota **(B)**, and colon mucosa microbiota **(C)** (*n* = 8). Bacterial community of ileal digesta **(D)**, colonic digesta **(E)**, and colon mucosa **(F)** at the phylum level. Bacterial community of ileal digesta **(G)**, colonic digesta **(H)**, and colon mucosa **(I)** at the genus level (abundance > 1%). M1, one meal per day; M3, three meals per day; M5, five meals per day.

The rarefaction curves plotting the number of sequences by the number of OTUs tended to approach the saturation plateau (Figure 1). As shown in Table 1, the species richness index (Chao 1) and the community diversity (Shannon) were not affected in the ileal digesta, colonic digesta, and colon mucosa. PCoA based on unweighted UniFrac distances showed that bacterial community composition in ileal digesta (Figure 2A), colonic digesta (Figure 2B), and colon mucosa (Figure 2C) were not affected by the different feeding frequencies.

**Table 1:** Richness estimators and diversity indices of the 16S rRNA gene libraries in ileal digesta, colonic digesta, and colon mucosa of pigs with different feeding frequencies^a^.

**Sample**	**Items**	**M1**	**M3**	**M5**	***P*-value**
Ileal digesta	Chao 1	222.57 ± 34.61	202.85 ± 27.29	238.03 ± 36.79	0.77
	Shannon	2.21 ± 0.21	2.43 ± 0.14	1.88 ± 0.17	0.12
Colonic digesta	Chao 1	1247.18 ± 50.16	1296.12 ± 56.61	1330.99 ± 66.44	0.60
	Shannon	4.94 ± 0.08	5.06 ± 0.11	5.07 ± 0.12	0.64
Colon mucosa	Chao 1	706.21 ± 19.20	694.13 ± 25.05	664.18 ± 29.81	0.49
	Shannon	4.34 ± 0.05	4.08 ± 0.09	4.06 ± 0.15	0.12

a *The richness estimators (Chao 1) and diversity indices (Shannon) were calculated. The data were analyzed by one-way ANOVA followed by Duncan's t-tests to evaluate differences in feeding regimens, data are expressed as mean, SEM, n = 8. OTUs, operational taxonomic units; M1, one meal per day; M3, three meals per day; M5, five meals per day*.

In the ileal digesta sample, Firmicutes, Proteobacteria, and Tenericutes were the dominant phyla, and no differences in the relative abundance were found (Figure 2D). At the genus level, the feeding regimen had no significant effect on the relative abundance of the 10 most dominating genera (>1%) (Figure 2G), except for *Turicibacter*, which was significantly higher in the M1 group than in the M5 group (Table 2). Firmicutes and Bacteroidetes were the dominant phyla in colonic digesta samples, and no differences in the relative abundance of ileum bacteria were found. In the colonic digesta sample, Firmicutes and Bacteroidetes were the dominant phyla, and no differences in the relative abundance were found (Figure 2E). At the genus level, the feeding regimen had no significant effect on the relative abundance of the 27 most dominating genera (>1%) (Figure 2H), except for *Phascolarctobacterium*, which was significantly lower in the M1 group than in the M5 group (Table 2). In the colon mucosa sample, Firmicutes and Bacteroidetes were the dominant phyla, and no differences in the relative abundance were found (Figure 2F). At the genus level, the feeding regimen had no significant effect on the relative abundance of the 25 most dominating genera (>1%) (Figure 2I), except for *Oscillibacter*, which was significantly higher in the M3 group than in the M5 group (Table 2).

**Table 2:** Relative abundance of microbial genera (percentage) significantly affected by feeding regimen in the ileal digesta, colonic digesta, and colon mucosa of pigs^1^.

**Sample**	**Genus**	**Group**			***P*-value**
		**M1**	**M3**	**M5**	
Ileal digesta	*Turicibacter*	8.52 ^a^	6.65 ^ab^	1.96 ^b^	0.03
Colonic digesta	*Phascolarctobacterium*	0.77 ^b^	1.02 ^ab^	1.85 ^a^	0.02
Colon mucosa	*Oscillibacter*	1.12 ^ab^	1.20 ^a^	0.69 ^b^	0.02

1 *Significantly changed bacteria in the ileal digesta, colonic digesta, and colon mucosa of pigs at the genus level (relative abundance > 1%)*.

a,b *Mean values within a line with different superscript letters differ significantly (p < 0.05)*.

*M1, one meal per day; M3, three meals per day; M5, five meals per day*.

At the dominant OTU level, M1 and M3 groups significantly increased the relative abundance of *Turicibacter sanguinis* and *Clostridium saudiense* in the ileal digesta than those in the M5 group (Supplementary Table 3). In colonic digesta (Supplementary Table 3), the feeding regimen had no significant effect on the relative abundance of the 18 most dominating OTU (>1%). In colon mucosa (Supplementary Table 4), the relative abundance of *Streptococcus pasteurianus* of pigs in the M3 group was higher than that of pigs in the M1 group.

In addition, concentrations of short-chain fatty acids (SCFAs), including total SCFA, acetate, propionate, butyrate, valerate, isovalerate, and isobutyrate in the colonic digesta among the three groups were no significant difference (Table 3).

**Table 3:** Concentrations of short-chain fatty acids (SCFAs) in the colonic digesta in pigs^a^.

**Items**	**M1**	**M3**	**M5**	***P*-value**
Total SCFA μmol/g digesta	71.39 ± 3.02	78.04 ± 3.21	70.33 ± 6.22	0.46
Acetate μmol/g digesta	33.65 ± 1.75	37.17 ± 1.90	33.99 ± 3.54	0.60
Propionate μmol/g digesta	26.83 ± 1.02	29.93 ± 1.68	25.85 ± 2.12	0.24
Butyrate μmol/g digesta	6.67 ± 0.47	6.88 ± 0.47	6.31 ± 0.71	0.78
Valerate μmol/g digesta	1.16 ± 0.17	0.95 ± 0.06	1.05 ± 0.16	0.58
Isovalerate μmol/g digesta	1.84 ± 0.10	1.78 ± 0.10	1.85 ± 0.19	0.94
Isobutyrate μmol/g digesta	1.25 ± 0.07	1.35 ± 0.06	1.28 ± 0.12	0.77

a *The data were analyzed by one-way ANOVA followed by Duncan's t-tests to evaluate differences in feeding regimens, data are expressed as mean ± SEM, n = 8*.

*M1, one meal per day; M3, three meals per day; M5, five meals per day*.

### Summary of RNA and Sequencing Quality Control

The present study established 24 cDNA libraries from ileum mucosa and colon mucosa of pigs in the three groups. For the ileum mucosa, after filtering low-quality reads and removal of ribosomal RNA reads, the clean reads rate of all samples was higher than 97%, the low-quality reads rate was lower than 1.5%. More than 95% of reads in each library were uniquely mapped to the pig genome (Sus scrofa 11.1) and the Q30 value of each library exceeded 93% (Table 4). For the colon mucosa, after filtering low-quality reads and removal of ribosomal RNA reads, the clean reads rate of all samples was higher than 97%, the low-quality reads rate was lower than 1.5%. More than 94% of reads in each library were uniquely mapped to the pig genome and the Q30 value of each library exceeded 93% (Table 4).

**Table 4:** Characteristics of the reads from ileum mucosa and colon mucosa tissues libraries.

**Sample ID^**a**^**	**Raw reads**	**Clean reads**	**Clean Reads Rate (%)**	**Low-quality Reads Rate (%)**	**Mapped reads**	**Mapping ratio (%)^**b**^**	**GC content**	**Q30 value (%)^**c**^**
I-M1-1	52,312,360	51,370,738	98.20	0.81	49,152,431	95.68	52.64	94.11
I-M1-2	50,058,777	48,957,484	97.80	0.97	46,759,664	95.51	53.29	94.33
I-M1-3	53,632,274	53,020,866	98.86	0.98	50,617,057	95.47	52.38	94.24
I-M1-4	55,803,518	54,910,662	98.40	0.87	52,629,635	95.85	51.97	94.12
I-M3-1	52,876,564	52,205,032	98.73	0.85	49,975,326	95.73	51.88	93.87
I-M3-2	54,503,146	53,402,182	97.98	1.04	51,093,197	95.68	51.68	94.14
I-M3-3	57,930,238	57,061,284	98.50	0.79	54,431,581	95.39	51.42	93.82
I-M3-4	57,176,084	56,444,230	98.72	0.83	53,762,734	95.25	52.69	94.43
I-M5-1	64,117,173	63,193,886	98.56	0.82	60,186,956	95.24	53.69	94.30
I-M5-2	58,166,415	56,723,888	97.52	1.12	54,240,433	95.62	52.23	94.16
I-M5-3	51,757,333	50,799,822	98.15	0.89	48,359,038	95.20	52.82	94.11
I-M5-4	49,401,461	48,423,312	98.02	0.92	46,002,569	95.00	52.00	93.70
C-M1-1	56,285,502	55,384,934	98.40	0.86	52,793,100	95.32	49.73	93.86
C-M1-2	42,670,787	41,646,688	97.60	1.18	39,851,000	95.69	51.03	94.19
C-M1-3	58,807,901	57,984,590	98.60	0.82	55,400,514	95.54	52.22	93.61
C-M1-4	61,005,484	60,334,424	98.90	0.96	57,782,461	95.77	51.85	93.71
C-M3-1	56,713,558	55,567,944	97.98	1.09	52,446,830	94.38	51.63	93.16
C-M3-2	61,775,714	60,941,742	98.65	0.75	58,097,898	95.33	50.99	93.57
C-M3-3	57,159,244	55,918,888	97.83	0.98	53,629,245	95.91	52.46	94.18
C-M3-4	65,630,497	64,724,796	98.62	0.69	61,775,967	95.44	51.46	93.78
C-M5-1	62,906,092	61,176,174	97.25	0.95	58,033,824	94.86	51.65	93.79
C-M5-2	59,493,049	58,731,538	98.72	0.84	55,961,326	95.28	51.46	93.52
C-M5-3	57,015,236	56,325,352	98.79	0.99	53,743,823	95.42	51.11	93.37
C-M5-4	58,609,111	57,495,538	98.10	1.01	54,684,743	95.11	51.87	94.16

a *I-: Ileum mucosa tissue; C-: Colon mucosa tissue; M1, one meal per day; M3, three meals per day; M5, five meals per day*.

b *Mapping ratio: mapped reads/clean reads*.

c *Q30 value: bases of Q ≥ 30/all bases of sequencing*.

### Transcriptomic Profiling of the Ileum Mucosa

**Figure 3:**
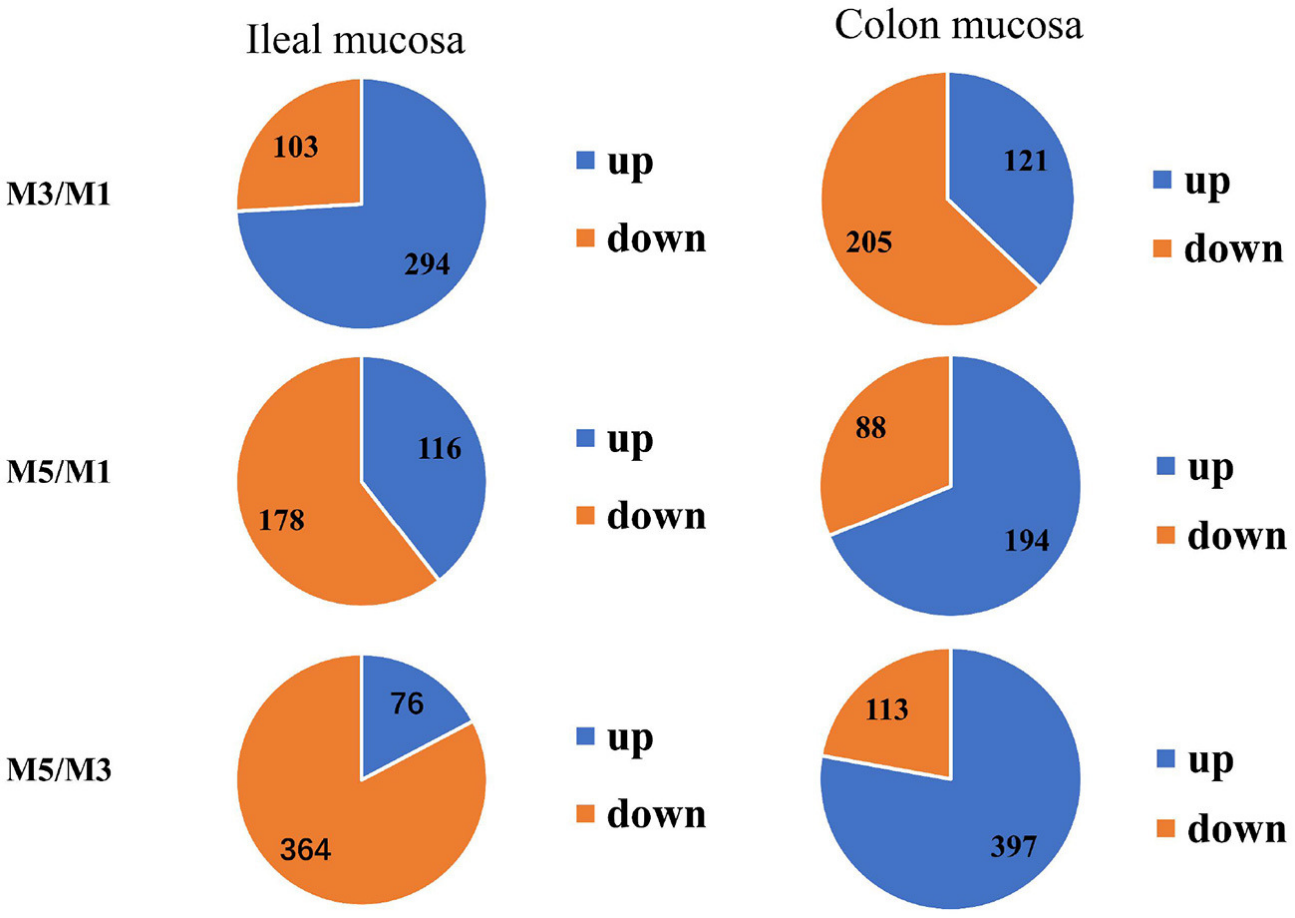
Numbers of the total differentially expressed genes as well as the up- and down-regulated genes in the ileum mucosa and colon mucosa of pigs among the one meal feeding daily (M1), three meals feeding daily (M3), and five meals feeding daily (M5) groups.

The genes of the ileum mucosa transcriptome profile with *P* < 0.05 and FC ≥ 1.5 or < 0.67 were considered as significant differences and were selected for further analysis. There were 397 DEGs between the M3 and M1 groups, 294 genes were up-regulated and 103 genes were down-regulated. There were 294 DEGs between the M5 and M1 groups, 116 genes were up-regulated and 178 genes were down-regulated. There were 440 DEGs between the M5 and M3 groups, 76 genes were up-regulated, and 364 genes were down-regulated (Figure 3).

**Figure 4:**
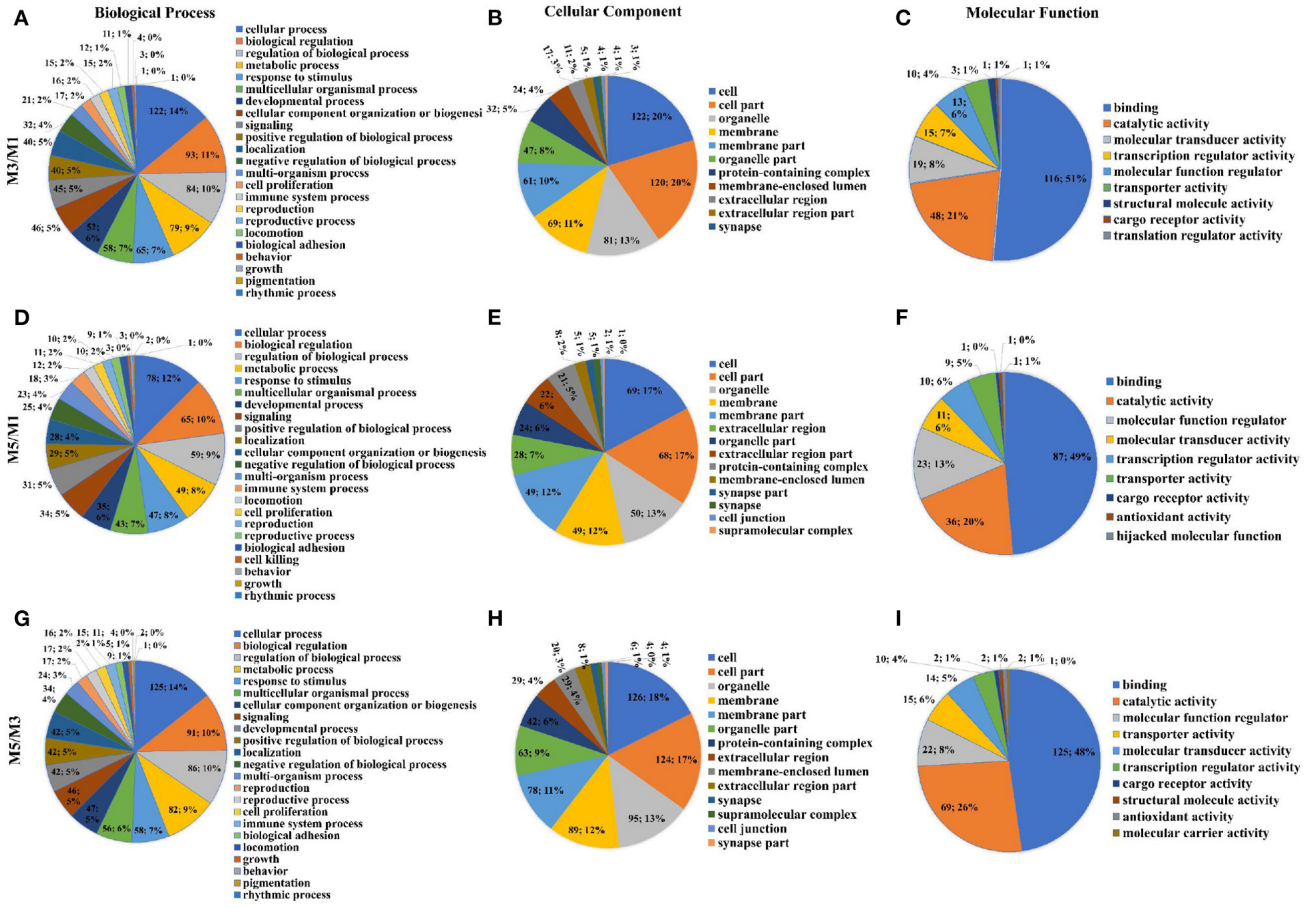
Proportions of different gene ontology (GO) terms (level two) in the ileum mucosa of pigs with different feeding regimens. Proportions of different GO biological process terms **(A)**, GO cellular component terms **(B)**, GO molecular function terms **(C)** between M3 and M1 groups. Proportions of different GO biological process terms **(D)**, GO cellular component terms **(E)**, GO molecular function terms **(F)** between M5 and M1 groups. Proportions of different GO biological process terms **(G)**, GO cellular component terms **(H)**, GO molecular function terms **(I)** between M5 and M3 groups. M1, one meal per day; M3, three meals per day; M5, five meals per day.

Analysis of the DEGs using level two GO putative terms showed similar proportions of the terms in the pairwise comparison between M3 and M1 groups, M5 and M1 groups, and M5 and M3 groups (Figure 4). In the pairwise comparison between M3 and M1 groups, M5 and M1 groups, and M5 and M3 groups, the types and proportions of genes associated with cellular components were highly similar, and with minor differences in proportion. The GO analysis of level two molecular function terms showed that genes putatively involved in binding and catalytic activity represented the largest (>69%) proportion of DEGs in the pairwise comparison between M3 and M1 groups, M5 and M1 groups, and M5 and M3 groups. In the pairwise comparison between M3 and M1 groups, M5 and M1 groups, and M5 and M3 groups, 23 different terms of level two biological process were detected, and the proportion was approximately equal. In which, those associated with the cellular process, biological regulation, regulation of the biological process, and metabolic processes were the most abundant.

**Figure 5:**
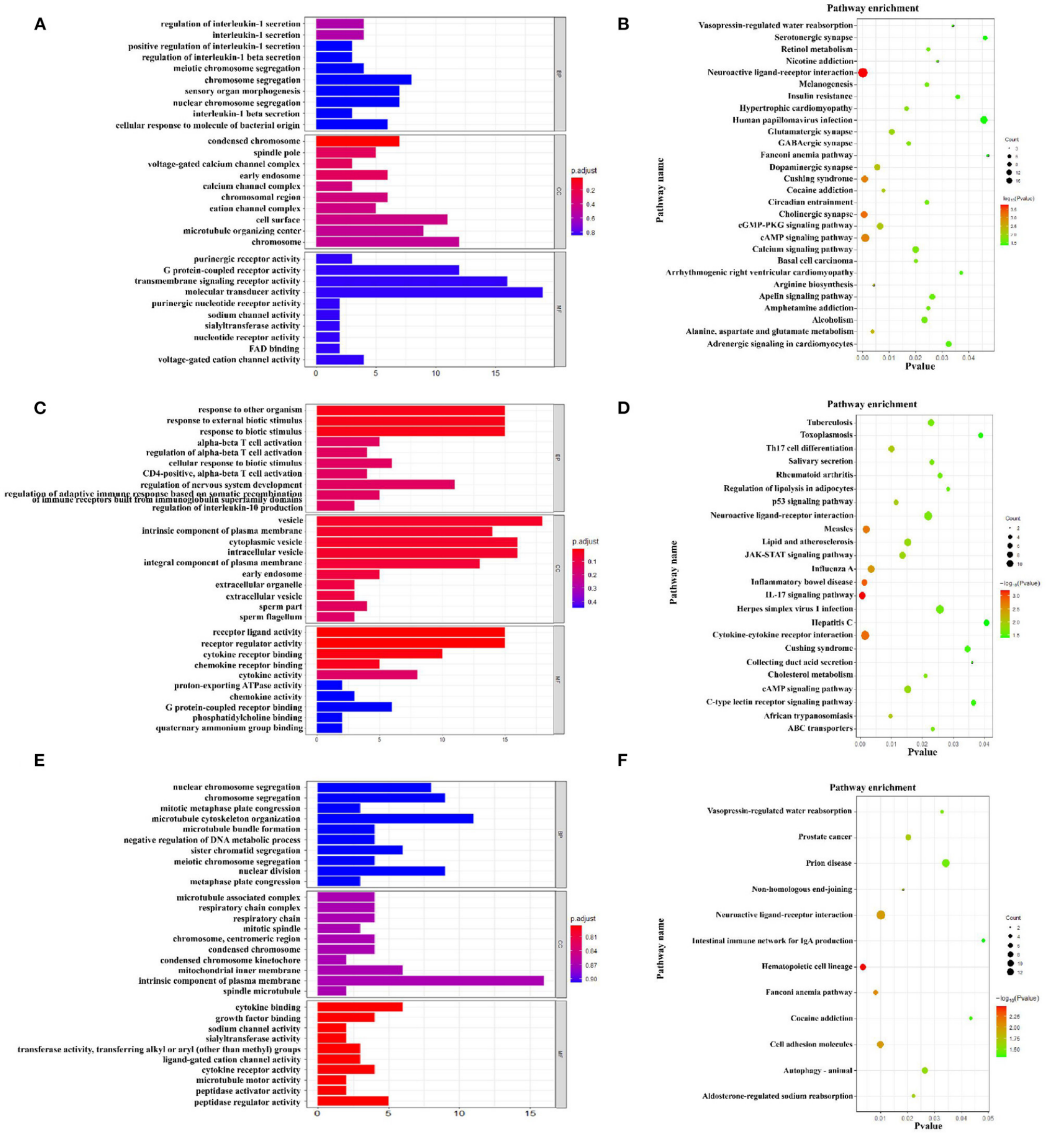
Function analysis of different expression genes (DEGs) in ileum mucosa between two treatment groups based on Gene Ontology and KEGG pathway. **(A)** Enriched GO terms of DEGs between M3 and M1 groups; **(B)** KEGG enrichment analysis of DEGs between M3 and M1 groups; **(C)** Enriched GO terms of DEGs between M5 and M1 groups; **(D)** KEGG enrichment analysis of DEGs between M5 and M1 groups; **(E)** Enriched GO terms of DEGs between M5 and M3 groups; **(F)** KEGG enrichment analysis of DEGs between M5 and M3 groups. M1, one meal per day; M3, three meals per day; M5, five meals per day.

Figures 5A,C,E showed the top 10 enriched GO terms of the three main functional categories (biological process, cellular components, and molecular function) in the pairwise comparison between M3 and M1 groups, M5 and M1 groups, and M5 and M3 groups, respectively. At the biological process level, most of the DEGs in ileum mucosa were significantly represented in GO terms of regulation of protein localization in the pairwise comparison between M3 and M1 groups, most of the DEGs were enriched in GO terms (response to other organisms, response to external biotic stimulus, and response to biotic stimulus) in the pairwise comparison between M5 and M1 groups, most of the DEGs were enriched in GO term of microtubule cytoskeleton organization in the pairwise comparison between M5 and M3 groups.

Next, the DEGs were subjected to pathway enrichment analysis base on the KEGG database (Sus scrofa). As for the pathway analysis, 28 KEGG pathways (*P* < 0.05) (Figure 5B), including neuroactive ligand-receptor interaction, cholinergic synapse, cushing syndrome, were significantly enriched in the pairwise comparison between M3 and M1 groups. In the pairwise comparison between M5 and M1 groups, DEGs were enriched in 24 KEGG pathways (Figure 5D), including IL-17 signaling pathway, inflammatory bowel disease, cytokine-cytokine receptor interaction. In the pairwise comparison between M5 and M3 groups, DEGs were enriched in 12 KEGG pathways (Figure 5F), including hematopoietic cell lineage, fanconi anemia pathway cell adhesion molecules.

### Transcriptomic Profiling of the Colon Mucosa

There were 326 differentially expressed genes between M3 and M1 groups, in which 121 genes were up-regulated and 205 genes were down-regulated. There were 282 differentially expressed genes between M5 and M1 groups, in which 194 genes were up-regulated and 88 genes were down-regulated. Finally, there were 510 differentially expressed genes were identified and annotated between M5 and M3 groups, in which 397 genes were up-regulated and 113 genes were down-regulated (Figure 3).

**Figure 6:**
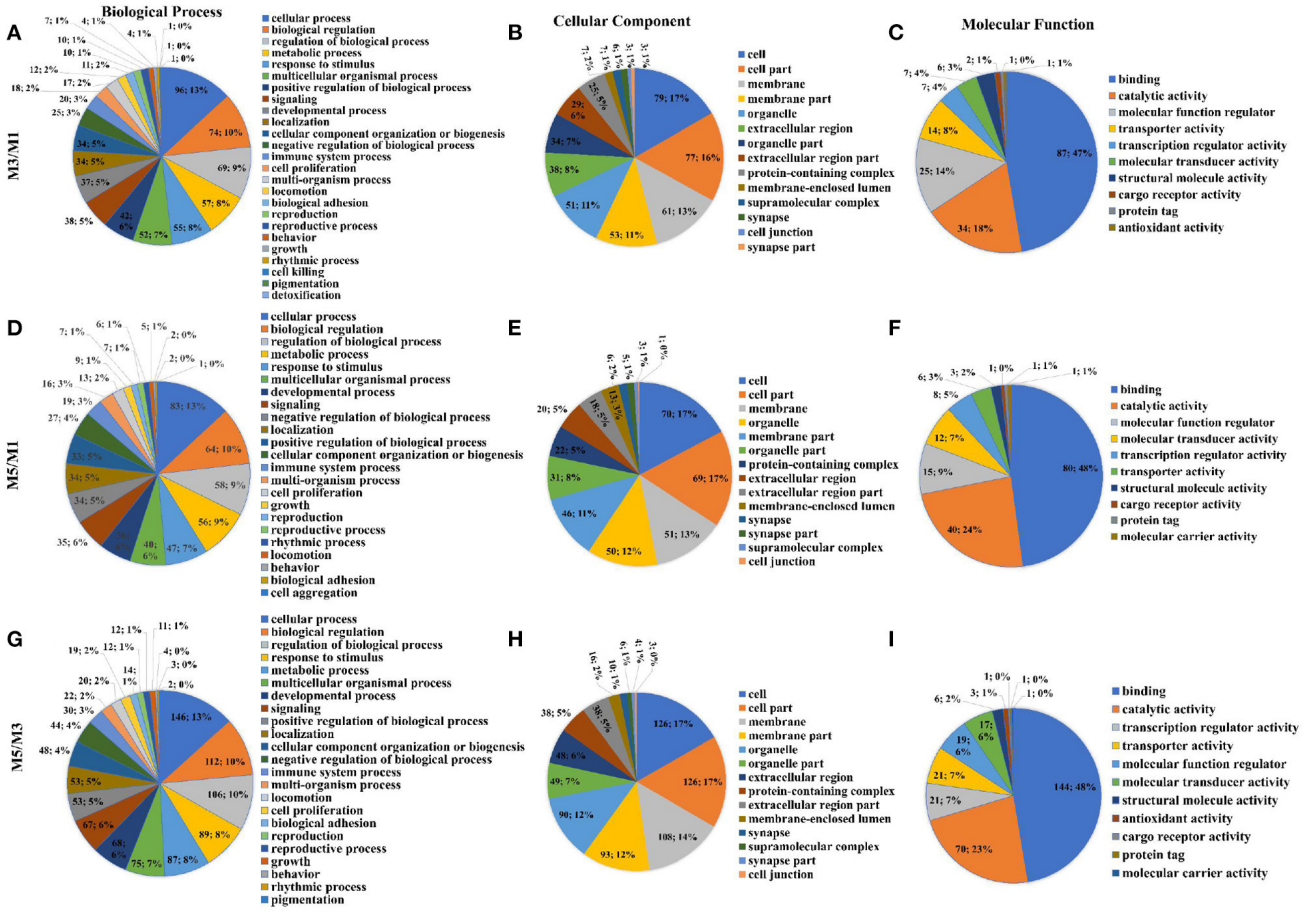
Proportions of different gene ontology (GO) terms (level two) in the colon mucosa of pigs with different feeding regimens. Proportions of different GO biological process terms **(A)**, GO cellular component terms **(B)**, GO molecular function terms **(C)** between M3 and M1 groups. Proportions of different GO biological process terms **(D)**, GO cellular component terms **(E)**, GO molecular function terms **(F)** between M5 and M1 groups. Proportions of different GO biological process terms **(G)**, GO cellular component terms **(H)**, GO molecular function terms **(I)** between M5 and M3 groups. M1, one meal per day; M3, three meals per day; M5, five meals per day.

Results of two GO putative terms showed similar proportions of the terms in the pairwise comparison between M3 and M1 groups, M5 and M1 groups, and M5 and M3 groups (Figure 6). In the pairwise comparison between M3 and M1 groups, M5 and M1 groups, and M5 and M3 groups, the types and proportions of genes associated with cellular components were highly similar, and with minor differences in proportion. The GO analysis of level two molecular function terms showed that genes putatively involved in binding and catalytic activity represented the largest (>65%) proportion of DEGs in the pairwise comparison between M3 and M1 groups, M5 and M1 groups, and M5 and M3 groups. In the pairwise comparison between M3 and M1 groups, M5 and M1 groups, and M5 and M3 groups, the proportions of genes associated with level two biological process were highly similar, and those major types were cellular process, biological regulation, regulation of the biological process, and metabolic processes.

**Figure 7:**
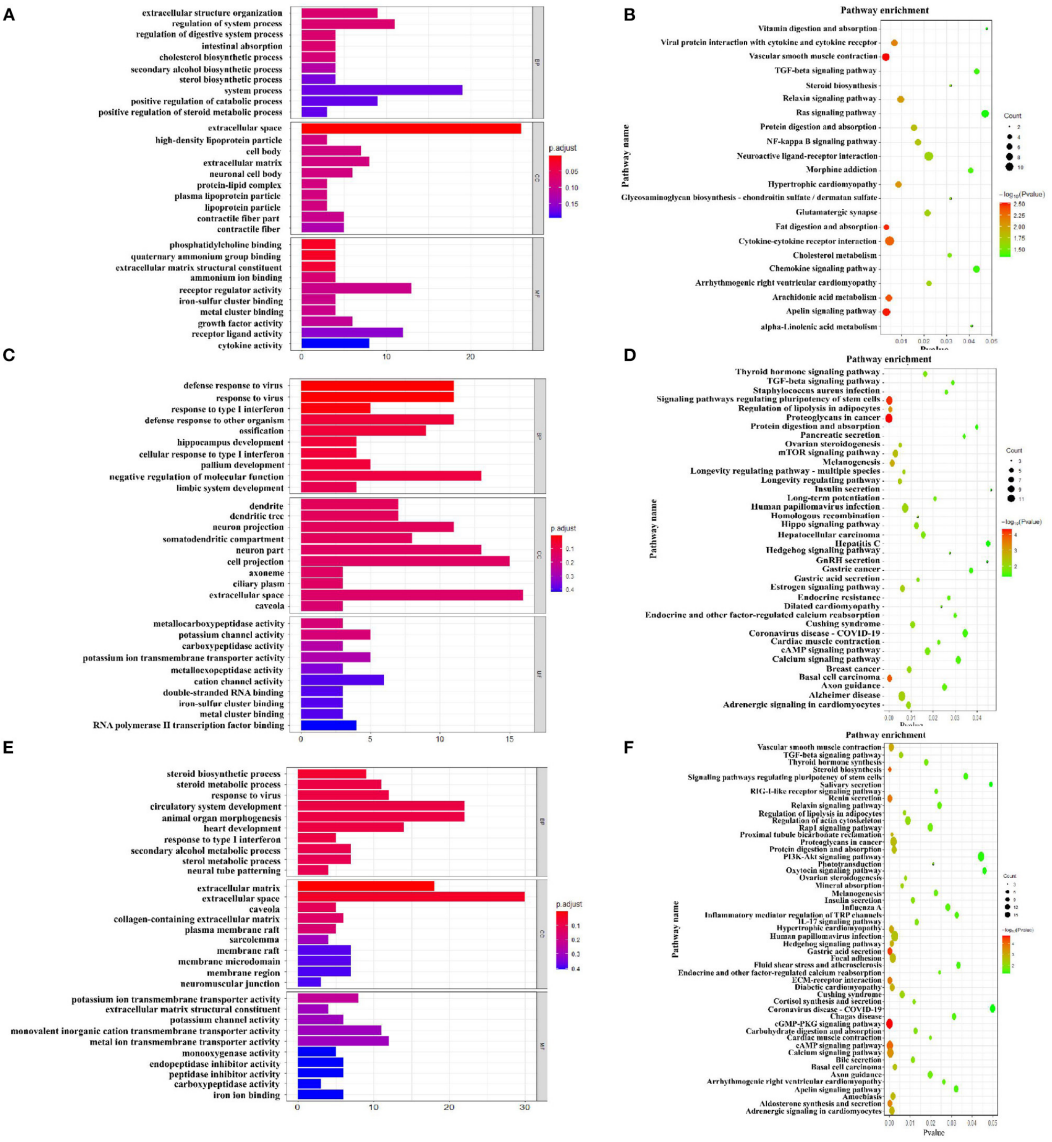
Function analysis of different expression genes (DEGs) in colon mucosa between two treatment groups based on Gene Ontology and KEGG pathway. **(A)** Enriched GO terms of DEGs between M3 and M1 groups; **(B)** KEGG enrichment analysis of DEGs between M3 and M1 groups; **(C)** Enriched GO terms of DEGs between M5 and M1 groups; **(D)** KEGG enrichment analysis of DEGs between M5 and M1 groups; **(E)** Enriched GO terms of DEGs between M5 and M3 groups; **(F)** KEGG enrichment analysis of DEGs between M5 and M3 groups. M1, one meal per day; M3, three meals per day; M5, five meals per day.

The Figures 7A,C,E showed the top 10 terms of the three main functional categories (biological process, cellular components, and molecular function) in the pairwise comparison between M3 and M1 groups, M5 and M1 groups, and M5 and M3 groups, respectively. At the biological process level, most of the DEGs in the colon mucosa were significantly represented in the term of regulation of system process in the pairwise comparison between M3 and M1 groups, the term of negative regulation of molecular function in the pairwise comparison between M5 and M1 groups, and the terms of circulatory system development, animal organ morphogenesis, and anatomical structure formation involved in morphogenesis in the pairwise comparison between M5 and M3 groups.

As for the pathway analysis, DEGs were significantly enriched in 22 KEGG pathways (*P* < 0.05) in the pairwise comparison between M3 and M1 groups (Figure 7B), including vascular smooth muscle contraction, apelin signaling pathway, fat digestion, and absorption. In the pairwise comparison between M5 and M1 groups, DEGs were enriched in 38 KEGG pathways (Figure 7D), including proteoglycans in cancer, signaling pathways regulating pluripotency of stem cells, basal cell carcinoma. In the pairwise comparison between M5 and M3 groups, DEGs were enriched in 51 KEGG pathways (Figure 7F), including the cGMP-PKG signaling pathway, gastric acid secretion, steroid biosynthesis.

### DEGs Related to Metabolism in Ileum Mucosa

Differentially expressed genes related to metabolism and their major metabolic types in ileum mucosa were summarized. As shown in Supplementary Table 5, 22 DEGs were involved in carbohydrate metabolism between M3 and M1 groups. Among these DEGs, two genes (*CHST13* and *SDHAF3*) were involved in the carbohydrate biosynthetic process and were up-regulated in the M3 group, while two genes (*SLC5A4* and *TRIB3*) were involved in carbohydrate transport and were down-regulated in the M3 group. Furthermore, 36 DEGs were involved in lipid metabolism. Among these DEGs, 11 genes were involved in the lipid biosynthetic process, seven (*ACSM4, FCER1A, GAL3ST4, PIK3C2G, PRKAA2, ST8SIA2*, and *St8sia4*) of which were up-regulated in the M3 group, while four genes (*DHCR24, FA2H, SREBF1*, and *TRIB3*) were down-regulated. Five DEGs were involved in the lipid catabolic process, three (*ACOX1, CLPS*, and *PLCL1*) of which were down-regulated in the M3 group, while two genes (*ADRB2* and *CRABP1*) were up-regulated. Six DEGs were involved in lipid transport, three of which (*ABCB11, ATP8B4*, and *GALR1*) were up-regulated in the M3 group, while three genes (*NOS2, OSBP2*, and *SYT7*) were down-regulated. The *ACOX1* gene was involved in lipid oxidation and was down-regulated in the M3 group. There were 23 DEGs involved in amino acid metabolism. Among these, the *SLC38A11* gene was involved in amino acid transport and was up-regulated in the M3 group.

The results further showed that 14 DEGs were involved in carbohydrate metabolism between M5 and M1 groups. Among these DEGs, the *AQP5* gene was involved in carbohydrate transport and was down-regulated in the M5 group. There were 23 DEGs were involved in lipid metabolism. Among these DEGs, eight genes were involved in the lipid biosynthetic process, five (*APOA1, APOC3, ACACB, FDPS*, and *IFNG*) of which were down-regulated in the M5 group, while three genes (*GAL3ST4, SPTSSB*, and *ST8SIA2*) were up-regulated. Five DEGs were involved in the lipid catabolic process, four (*ACACB, APOA1, APOC3*, and *PLCE1*) of which were down-regulated in the M5 group, while the *PLCH2* was up-regulated. Four DEGs (*ABCA1, APOA1, APOC3*, and *SLC10A6*) were involved in lipid transport and were down-regulated in the M5 group. There were 19 DEGs involved in amino acid metabolism. Among these DEGs, the *HAL* gene was involved in the cellular amino acid metabolic process and was up-regulated in the M5 group, the *SLC6A18* gene was involved in amino acid transport and was up-regulated in the M5 group.

Between M5 and M3 groups, 15 DEGs were involved in carbohydrate metabolism. Among these DEGs, the *SLC2A6* gene was involved in carbohydrate transport and was up-regulated in the M5 group, while the *RHOQ* gene was down-regulated. The *PGM2L1* gene was involved in the carbohydrate catabolic/biosynthetic process and was down-regulated in the M5 group. There were 22 DEGs involved in lipid metabolism. Among these DEGs, six genes were involved in the lipid biosynthetic process, 5 (*FDPS, St8sia4, ACSL4, PTGDS*, and *SCD5*) of which were down-regulated in the M5 group, while the *SMPD1* gene was up-regulated. Three DEGs were involved in the lipid catabolic process, two (*ENPP7* and *PLCL1*) of which were down-regulated in the M5 group, while the *SMPD1* gene was up-regulated. Five DEGs (*ABCA1, ACSL4, ATP8B4, GALR1*, and *ITGAV*) were involved in lipid transport and were down-regulated in the M5 group. There were 32 DEGs involved in amino acid metabolism. Among these, the *SARDH* gene was involved in the cellular amino acid catabolic process and was up-regulated in the M5 group. Three DEGs were involved in amino acid transport, two (*SLC38A11* and *TRPC4*) of which were down-regulated in the M5 group, while the *SLC7A4* gene was up-regulated.

### DEGs Related to Metabolism in Colon Mucosa

As shown in Supplementary Table 5, 23 DEGs were involved in carbohydrate metabolism between M3 and M1 groups. Among these DEGs, the *CHST13* gene was involved in the carbohydrate biosynthetic process and was up-regulated in the M3 group, while the *SHAS2* gene was down-regulated. The *SLC5A11* gene was involved in carbohydrate transport and was up-regulated in the M3 group. There were 25 DEGs involved in lipid metabolism. Among these DEGs, seven genes were involved in the lipid biosynthetic process, four (*APOA1, BMP5, HSD17B7*, and *PTGDS*) of which were down-regulated in the M3 group, while three genes (*APOA2, APOA4*, and *PRKAA2*) were up-regulated. Five DEGs were involved in the lipid catabolic process, three (*APOA1, PLA2G12A*, and *PLA2G2C*) of which were down-regulated in the M3 group, while two genes (*APOA2* and *APOA4*) were up-regulated. Seven DEGs were involved in lipid transport, two of which (*APOA2* and *APOA4*) were up-regulated in the M3 group, while five genes (*APOA1, ATP10B, LBP, PLA2G12A*, and *PLA2G2C*) were down-regulated. There were 21 DEGs involved in amino acid metabolism. Among these, two genes (*SLC1A1* and *TRPC4*) were involved in amino acid transport and were up-regulated in the M3 group.

Between M5 and M1 groups, 14 DEGs were involved in carbohydrate metabolism between M5 and M1 groups. Among these DEGs, the *FFAR3* gene was involved in carbohydrate transport and was up-regulated in the M5 group. The *CHST13* gene was involved in the carbohydrate biosynthetic process and was up-regulated in the M5 group. There were 27 DEGs involved in lipid metabolism. Among these DEGs, nine genes (*ACSM4, AGMO, ATM, CDS2, INSIG1, NPC1L1, PRKAA2, SPTLC3*, and *St8sia4*) were involved in the lipid biosynthetic process and were up-regulated in the M5 group. The *ADRB2* gene was involved in the lipid catabolic process and was up-regulated in the M5 group. Four DEGs (*NPC1L1, PLN4, PTCH1*, and *SYT7*) were involved in lipid transport and were up-regulated in the M5 group.

The results further showed that 25 DEGs were involved in carbohydrate metabolism between M5 and M3 groups. Among these DEGs, the *AQP1* gene was involved in carbohydrate transport and was up-regulated in the M5 group, while the *SLC2A2* gene was down-regulated. The *MLXIPL* gene was involved in the carbohydrate catabolic process and was down-regulated in the M5 group. The *SHAS2* gene was involved in the carbohydrate biosynthetic process and was down-regulated in the M5 group. There were 40 DEGs involved in lipid metabolism. Among these DEGs, 13 genes were involved in the lipid biosynthetic process, 12 (*AGT, CDS2, FDFT1, FGFR4, HSD17B7, INSIG1, MLXIPL, PTGIS, SLC27A2, SPTLC3, sqle*, and *STAR*) of which were up-regulated in the M5 group, while the *CYP11A1* gene was down-regulated. The *SLC27A2* gene was involved in the lipid catabolic process and was up-regulated in the M5 group, while the *LIPE* gene was down-regulated. Nine DEGs (*ABCB11, AGT, PLIN4, PRELID2, PTCH1, SLC27A2, STAR, SYT7*, and *TNFAIP8L3*) were involved in lipid transport and were up-regulated in the M5 group.

### Validation of RNA-Seq Results by qRT-PCR

**Figure 8:**
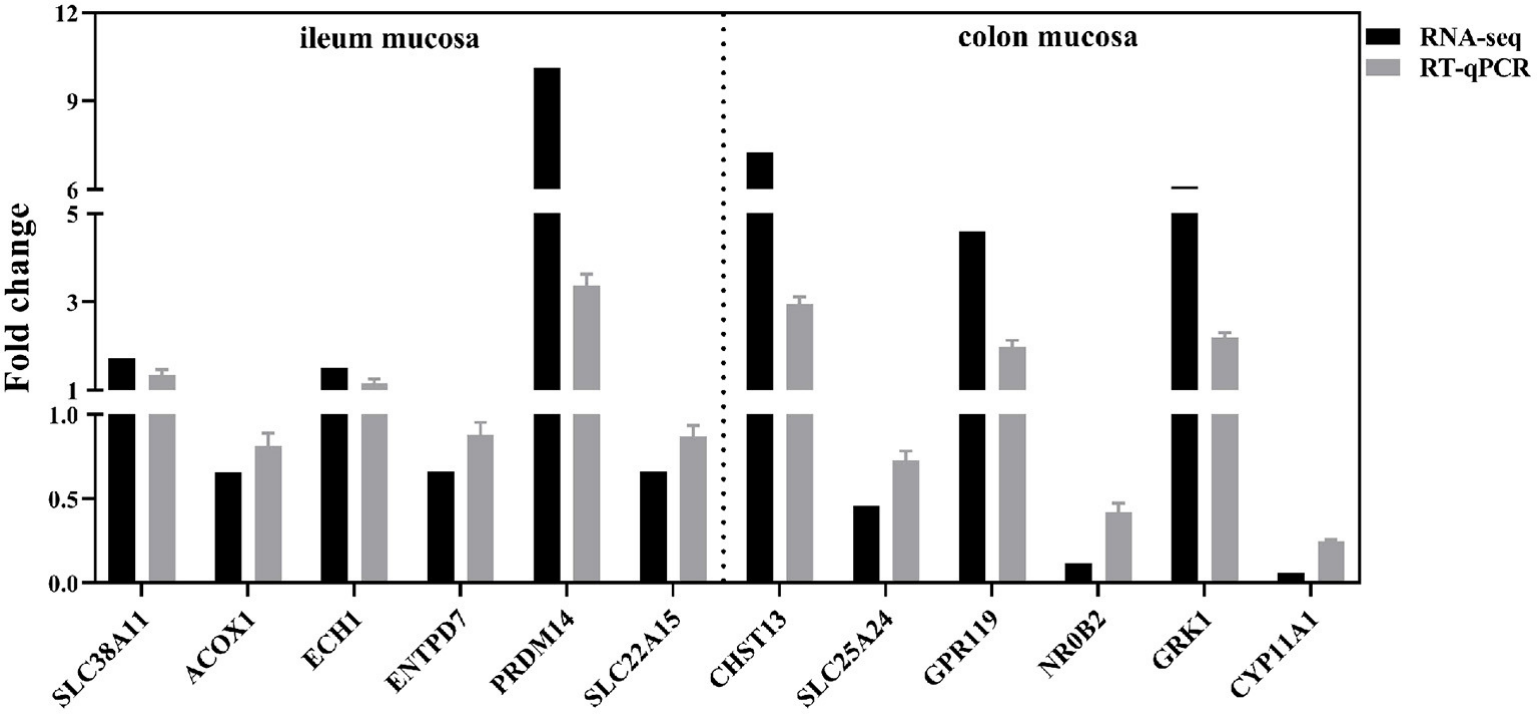
Quantitative real-time PCR validation of the selected DEGs. *SLC38A11*, solute carrier family 38 member 11; *ACOX1*, acyl-CoA oxidase 1; *ECH1*, enoyl-CoA hydratase 1; *ENTPD7*, ectonucleoside triphosphate diphosphohydrolase 7; *PRDM14*, PR/SET domain 14; *SLC22A15*, solute carrier family 22 member 15; *CHST13*, carbohydrate sulfotransferase 13; *SLC25A24*, solute carrier family 25 member 24; *GPR119*, G protein-coupled receptor 119; *NR0B2*, nuclear receptor subfamily 0 group B member 2; *GRK1*, G protein-coupled receptor kinase 1; *CYP11A1*, cytochrome P450 family 11 subfamily A member 1. The relative quantification of the DEGs was calculated with the formula 2^−ΔΔCt^ and normalized to β-actin control (*n* = 8 per group).

To validate the transcriptomic results by qRT-PCR, six up-regulated genes (*SLC38A11, ECH1, PRDM14, CHST13, GPR119*, and *GRK1*) and six down-regulated genes (*ACOX1, ENTPD7, SLC22A15, SCL25A24, NR0B2*, and *CYP11A1*) were validated. As shown in Figure 8, the results showed the expression profiles of these genes detected by qRT-PCR were consistent with those detected by transcriptome, which confirmed the reliability of our RNA sequencing data.

## Discussion

In the present study, high-throughput sequencing technology was adapted to investigate the effects of different feeding frequencies per day on gene expression in ileum mucosa and colon mucosa, the microbial composition of ileal digesta, colonic digesta, and colon mucosa, as well as the microbial metabolites of colonic digesta of growing pigs. Results showed that the intestinal bacterial composition and the concentrations of SCFA in colonic digesta were not significantly affected by different feeding frequencies for 1 month. However, the DEGs in the intestinal mucosa transcriptome indicated that different feeding regimens affected the intestinal function of growing pigs.

As an important host genome, the intestinal microbiota plays an important role in maintaining physiological homeostasis and has become one of the fastest evolving research areas in recent years (22). The intestinal microbiota itself follows diurnal oscillations in composition and function whose regulation is driven by host eating rhythm. Studies have found that mealtime is now as important as the composition of the diet in entraining microbiome rhythmicity (12). For example, high-fat restricted feeding during the active phase caused some microbiota changes that were similar to those of normal diet feeding (23), late-night eating led to the impairment of gut barrier function and altered the diversity and composition of the gut microbiota (24), intermittent fasting shapes the intestinal microbiota in a daily fasting hour dependent manner (25). However, no significant difference was found in the three groups in our study in the diversity indices, richness estimators, and composition of microbiota from the anterior ileal digesta, colonic digesta, and colon mucosa except for *Turicibacter* at the genus level of the ileal digesta, *Phascolarctobacterium* at the genus level of the colonic digesta and *Oscillibacter* at the genus level of the colon mucosa (relative abundance > 1%).

The intestinal barrier is tightly regulated by the intestinal microbiota and metabolites. The dysfunction of the intestinal barrier caused by wrong time eating is related to intestinal dysbiosis, and a decrease in the level of colonic metabolite butyrate, a known intestinal barrier stabilizer (26, 27). The study of Li et al. (25) found that fasting for 16 h per day for 30 days led to significantly increased abundance of *Akkermansia*, which was associated with metabolic improvements including decreased liver triglyceride accumulation and alleviated intestinal inflammation (28), and significantly decreased the abundance of *Alistipes*, which might improve intestinal inflammation (29). These findings suggest that the effect of irregular eating patterns on health is likely to be linked to intestinal microbiota composition and their metabolites. In our study, the levels of SCFAs in the colonic digesta among the three groups were no significant differences. Part of the reason for the inconsistent results may be the intake behavior of pigs, the experimental design, and the sample collection in our study. First, the intake behavior of pigs is different from that of human beings and mice. When fed with adequate feed, pigs do not finish eating in a short time and continue to feed. Although the intake behaviors of the three groups of pigs in our study were different, the residual content in the gastrointestinal tract could be the reason that no significant difference was found in the composition of intestinal microbiota. Second, a simple T-cannula was not surgically applied to the growing pigs in this experiment, and the sample collection was made from 08:00 a.m. to 10:00 a.m. The diurnal oscillation of the intestinal microbiota within 24 h needs further study.

The expression profiles of randomly selected genes detected by qRT-PCR were consistent with those detected by transcriptome, which confirms the reliability of RNA sequencing data. In the present study, the distribution of DEGs in major functional terms (GO terms) for categories of biological processes were related to immune and inflammation functions of the ileum in the pairwise comparison between M3 and M1 groups, as well as M5 and M1 groups. Moreover, immune-related signaling pathways, such as *Th17* cell differentiation, inflammatory bowel disease, and IL-17 signaling pathway were enriched in the ileum in the pairwise comparison between the M5 and M1 groups. Although the DEGs in GO terms for categories biological process were not related to the immune and inflammation functions of the ileum in the pairwise comparison between M5 and M3 groups, the intestine immune network for IgA production pathway was enriched. As for the colon mucosa, the NF-kappa B signaling pathway was enriched in the pairwise comparison between M3 and M1 groups, IL-17 signaling pathway was enriched in the pairwise comparison between M5 and M3 groups. Studies have shown that fasting can reduce inflammation, however, its effect on the immune system is still elusive (30). Several observational and causal studies have shown that the gut and its microbial contents could be the main contributor to chronic low-grade inflammation linked with obesity (31, 32). Our results indicated that the metabolic disorder caused by irregular eating patterns was related to the influence of intestinal immune function, which was consistent with previous results (24).

Moreover, KEGG pathway enriched analysis showed nutrient digestion and absorption, such as arginine biosynthesis and alanine, aspartate, and glutamate metabolism pathways were affected in the ileum of pigs in the pairwise comparison between M3 and M1 groups. The pathways of regulation of lipolysis in adipocytes and cholesterol metabolism were enriched in the ileum of pigs in the pairwise comparison between M5 and M1 groups. As for the colon mucosa, the distribution of DEGs in GO terms for categories biological process were related to the metabolism functions of pigs in the pairwise comparison between M3 and M1 groups or M5 and M3 groups. Moreover, the pathways of vitamin digestion and absorption, steroid biosynthesis, protein digestion and absorption, fat digestion and absorption, cholesterol metabolism, arachidonic acid metabolism, and alpha-Linolenic acid metabolism were enriched in pigs in the pairwise comparison between the M3 and M1 groups. The pathways of regulation of lipolysis in adipocytes and protein digestion and absorption were enriched in the pairwise comparison between the M5 and M1 groups. The pathways of steroid biosynthesis, regulation of lipolysis in adipocytes, and protein digestion and absorption were enriched of pigs in the pairwise comparison between the M5 and M3 groups. The effect of different feeding regimens on vitamin and amino acid metabolism in the small intestine was also consistent with our previous study (33). Results of mucosa transcriptomic profiling indicated irregular eating patterns main affected the digestion and absorption of protein and lipids in diets, which is consistent with our previous study that the apparent digestibility of crude protein in the M3 group was significantly higher than that in the M1 group (7). The final body weight, average daily gain in M3 and M5 groups were significantly higher than in the M1 group while feeding, while gain was significantly lower than in the M1 group (7), partly due to the effect of feeding frequency on the digestion and absorption of protein and lipid.

The food we eat is digested in the gastrointestinal tract and its nutrients are absorbed into the body for various life activities. In the present study, analysis of DEGs related to the metabolism of carbohydrate, lipid, and amino acid in the intestine indicated feeding frequency significantly affected nutrient digestion and absorption. Compared with the M1 group, carbohydrate transport in the ileum of M3 and M5 groups was suppressed, partly due to the activity of duodenum sucrase in M3 and M5 groups was significantly decreased compared with the M1 group (*P* < 0.05), and the activity of duodenum maltase in the M5 group was significantly decreased compared with the M1 group (*P* < 0.05) (7), which reduced the hydrolysis capacity of carbohydrates in M3 and M5 groups. Carbohydrate transport in the ileum of the M5 group was enhanced compared with the M3 group, while in the colon of the M5 group was suppressed compared with the M3 group. Amino acid transport in the ileum and colon was enhanced in the M3 group compared with the M1 group. Moreover, the cellular amino acid catabolic process in the ileum of the M5 group was enhanced compared with the M1 and M3 groups, which may be related to the activities of trypsin and chymotrypsin in the pancreas of M3 and M5 groups were significantly increased (*P* < 0.05) (7).

Lipid biosynthetic and catabolic processes in the ileum were suppressed with the increase of feeding frequency, however, lipid biosynthetic and catabolic processes in the colon were enhanced with the increase of feeding frequency. The Colipase (*CLPS*) is a protein co-enzyme required for optimal enzyme activity of pancreatic lipase, which was down-regulated in the M3 group compared with the M1 group. The low expression of *CLPS* in the ileum of the M3 group reduced the hydrolysis of triglycerides by lipase and reduced the inhibitory effect of bile salts on the lipase-catalyzed intraduodenal hydrolysis of dietary long-chain triglycerides. The sterol regulatory element-binding transcription factor 1 (*SREBF1*) could regulate genes related to lipid and cholesterol production and its activity is regulated by sterol levels in the cell (34), which was down-regulated in the M3 group compared with the M1 group. Compared with the M1 group, the lower expression of *CLPS* and *SREBF1* in the ileum of the M3 group indicated lipid catabolism and biosynthesis were suppressed. The Niemann-Pick C1-Like 1 (*NPC1L1*) gene was involved in the digestion, absorption, and transport of lipid, which was up-regulated in the colon of the M5 group compared with the M1 group. Compared with the M1 group, the higher expression of *NPC1L1* in the M5 group increased cholesterol absorption.

The present study showed the transcriptomic responses in ileum mucosa and colon mucosa, and the microbial composition of ileal digesta, colonic digesta, and colon mucosa of growing pigs by different feeding frequencies. The influence of individual variations in growing pigs on intestinal microbiota and transcriptome profile cannot be ignored. Therefore, it is necessary to increase the number of replicates in each group to explore the response of feeding frequency to microbial composition and mucosal transcriptome profile. In addition, some studies are needed in the future to obtain more information about feeding frequency to microbial composition and mucosal transcriptome profile, including a simple T-cannula with pigs to explore the diurnal oscillation of the intestinal microbiota within 24 h, and the effect of different feeding regimens on pigs with different diet composition.

## Conclusions

In summary, the present study showed that under the condition of the same daily feed intake, different feeding frequencies had no significant effect on microbial composition, while significantly affected the expression of metabolism-related genes in the ileum and colon. Carbohydrate transport in the ileum of the M5 and M3 groups was suppressed, while that in the colon of the M5 and M3 groups was enhanced. Amino acid catabolism in the ileum of the M5 group was enhanced compared with the M1 and M3 groups. Lipid biosynthetic and catabolic processes in the ileum were suppressed with the increase of feeding frequency, however, those in the colon were enhanced with the increase of feeding frequency. These findings support the idea that meal frequency as a strategy affecting growth performance may be associated with the digestion and absorption of nutrients in the intestine.

## Data Availability Statement

The RNA-seq datasets were deposited into the GenBank Sequence Read Archive database under accession number PRJNA747849 and PRJNA747852. The raw reads of MiSeq sequencing of 16S ribosomal ribonucleic acid (rRNA) gene were deposited into the GenBank Sequence Read Archive database under accession number PRJNA748604, PRJNA748609, and PRJNA748605.

## Ethics Statement

The animal study was reviewed and approved by Animal Care and Use Committee of Nanjing Agricultural University in Nanjing, Jiangsu, China [ethic code: SYXK (SU) 2017-0007].

## Author Contributions

YS conceived and designed the study and guided on the paper flow and structure. HZ and PX conducted the animal experiment. HZ, LF, and MJ conducted the research. HZ wrote the manuscript. All authors had read and approved the final manuscript.

## Funding

This work was supported by the National Natural Science Foundation of China (31872362 and 32072688) and the National Key R&D Program of China (2018YFD0500404).

## Conflict of Interest

The authors declare that the research was conducted in the absence of any commercial or financial relationships that could be construed as a potential conflict of interest.

## Publisher's Note

All claims expressed in this article are solely those of the authors and do not necessarily represent those of their affiliated organizations, or those of the publisher, the editors and the reviewers. Any product that may be evaluated in this article, or claim that may be made by its manufacturer, is not guaranteed or endorsed by the publisher.
