# Supplementary material for: Feeding Frequency Modulates the Intestinal Transcriptome Without Affecting the Gut Microbiota in Pigs With the Same Daily Feed Intake
Source: Front Nutr. 2021 Oct 29;8:743343. doi: 10.3389/fnut.2021.743343 (PMC8589026; doi:10.3389/fnut.2021.743343)
Supplement: Supplementary file 1 [file Table_1.docx]

Supplementary Material

## Supplementary Tables

Table S1. Composition and nutrient level of the diet (air-dry basis).

| **Ingredient** | **Percentage (%)** | **Nutritional Compositions (%)** |  |
| --- | --- | --- | --- |
| Corn | 70.0 | Digestive energy（MJ/kg） | 14.60 |
| Soybean meal | 18.0 | Crude protein | 16.00 |
| Wheat bran | 6.50 | Lysine | 1.23 |
| Soybean oil | 1.90 | Methionine + Cystine | 0.70 |
| Lysine | 0.69 | Threonine | 0.79 |
| Methionine | 0.24 | Tryptophan | 0.22 |
| Threonine | 0.30 |  |  |
| Tryptophan | 0.07 |  |  |
| Calcium hydrogen phosphate | 0.45 |  |  |
| Stone powder | 0.50 |  |  |
| Salt | 0.30 |  |  |
| Multivitamins ^1^ | 0.03 |  |  |
| Minerals ^2^ | 0.20 |  |  |
| Choline chloride (50%) | 0.12 |  |  |
| Zeolite powder | 0.60 |  |  |
| Antioxidant | 0.05 |  |  |
| Antifungal agent | 0.05 |  |  |
| Total | 100.0 |  |  |

^1^ The mineral supply per kg diet was as follows: Fe 165 mg, Zn 165 mg, Cu 16.5 mg, Mn 30 mg, Co 0.15 mg, I 0.25 mg, and Se 0.25 mg. ^2^ The multivitamin supply per kg diet was as follows: VA 11,000 IU, VD3 1000 IU, VE 16 IU, VK1 1 mg, VB1 0.6 mg, VB2 0.6 mg, d-pantothenic acid 6 mg, nicotinic acid 10 mg, VB12 0.03 mg, folic acid 0.8 mg, and VB6 1.5 mg.

Table S2. Description of primers used in the RT-qPCR analysis of gene expression.

| **Items** | **Gene** | **Forward** | **Reverse** |
| --- | --- | --- | --- |
| Target | SLC38A11 | CTTTCCACCATTGCCTTTACTC | GCAAATATCCAGGCATCTTCTG |
|  | ACOX1 | CCTGATCGAAGCCTATAGACTG | CTGAATCTGGTGGAGTTTTTCG |
|  | ECH1 | GCACCTCCATAATCTCATCTCC | GGCTCTGGTTTCCAATGATTTT |
|  | ENTPD7 | TACTGTACAGAGGATGTGTTGC | CTCTGAGTGAGTACTGACCAAG |
|  | PRDM14 | CAGTGGATGCTTCTCTGCTAC | TTCTCTCTTGGTTCACGAGTTT |
|  | SLC22A15 | CTGTCTGGCCTCATAGAGATTC | CGAGGTGTAGATATAGACCACG |
|  | CHST13 | GAGTTTCTGGCTTACCTGCT | GTCTCGAACTTGCCCACTAC |
|  | SLC25A24 | GAGTTGATTCTCCGAAGCATTG | TCATGACTTTCAGACGATCCAA |
|  | GPR119 | CGTTCTTTATCACGAGCATTGT | ATAGGCATAGATGAGTGGGTTG |
|  | NR0B2 | TGATGTTCCAGGCCTCTATG | TGTCAACATCTCCAATGACAGG |
|  | GRK1 | ATGGTGGAGAAGAAGGTTCTG | GTAGATGTGGTACCTTAGGTCC |
|  | CYP11A1 | TTAAGGCCAATGTTACCGAGAT | CCATCTCATACAAGTGCCATTG |
| Reference | β-actin | CCACGAAACTACCTTCAACTC | TGATCTCCTTCTGCATCCTGT |

Table S3 Relative abundance of microbial OTUs (percentage) in the ileal digesta and colonic digesta of piglets under different feeding frequencies (n = 8) ^1^.

| Sample | OTU Name | M1 | M3 | M5 | *P*-value | Annotation |
| --- | --- | --- | --- | --- | --- | --- |
| Ileal digesta | OTU1 | 0.040 | 0.136 | 0.237 | 0.336 | *Escherichia fergusonii* (100%) |
|  | OTU2 | 0.176 | 0.126 | 0.015 | 0.797 | *Clostridium saudiense* (98.02%) |
|  | OTU3 | 0.083 ^a^ | 0.065 ^ab^ | 0.019 ^c^ | 0.034 | *Turicibacter sanguinis* (99.07%) |
|  | OTU4 | 0.062 | 0.063 | 0.038 | 0.339 | *Romboutsia timonensis* (100%) |
|  | OTU5 | 0.090 | 0.064 | 0.009 | 0.887 | *Clostridium saudiense* (98.27%) |
|  | OTU6 | 0.001 | 0.003 | 0.004 | 0.457 | *Streptococcus pasteurianus* (99.77%) |
|  | OTU7 | 0.004 | 0.004 | 0.011 | 0.773 | *Lactobacillus johnsonii* (100%) |
|  | OTU8 | 0.015 | 0.015 | 0.004 | 0.264 | *Terrisporobacter petrolearius* (99.26%) |
|  | OTU12 | 0.001 | 0.004 | 0.006 | 0.140 | *Actinobacillus indolicus* (97.44%) |
|  | OTU13 | 0.000 | 0.031 | 0.006 | 0.277 | *Actinobacillus indolicus* (97.44%) |
|  | OTU14 | 0.000 | 0.022 | 0.005 | 0.268 | *Actinobacillus minor* (99.30%) |
|  | OTU17 | 0.004 | 0.008 | 0.003 | 0.539 | *Mycoplasma sualvi* (100%) |
|  | OTU18 | 0.000 | 0.001 | 0.002 | 0.287 | *Streptococcus pasteurianus* (100%) |
|  | OTU19 | 0.026 ^a^ | 0.007 ^ab^ | 0.001 ^c^ | 0.028 | *Clostridium saudiense* (100%) |
|  | OTU22 | 0.000 | 0.004 | 0.006 | 0.235 | *[Actinobacillus] rossii* (97.20%) |
|  | OTU23 | 0.000 | 0.004 | 0.006 | 0.339 | *[Actinobacillus] rossii* (96.97%) |
|  | OTU26 | 0.004 | 0.002 | 0.000 | 0.277 | *Clostridium butyricum* (100%) |
|  | OTU28 | 0.002 | 0.003 | 0.001 | 0.572 | *Mycoplasma sualvi* (99.77%) |
| Colonic digesta | OTU6 | 0.015 | 0.019 | 0.017 | 0.539 | *Streptococcus pasteurianus* (99.77%) |
|  | OTU7 | 0.009 | 0.005 | 0.007 | 0.756 | *Lactobacillus johnsonii* (100%) |
|  | OTU9 | 0.021 | 0.021 | 0.029 | 0.939 | *Prevotella copri DSM* (97.88%) |
|  | OTU11 | 0.004 | 0.038 | 0.018 | 0.192 | *Prevotella shahii* (89.62%) |
|  | OTU10 | 0.011 | 0.019 | 0.011 | 0.724 | *Muribaculum intestinale* (88.68%) |
|  | OTU15 | 0.000 | 0.000 | 0.000 | 0.152 | *Megasphaera elsdenii* (100%) |
|  | OTU16 | 0.013 | 0.020 | 0.028 | 0.259 | *Prevotella oralis* (92.92%) |
|  | OTU18 | 0.010 | 0.012 | 0.005 | 0.022 | *Streptococcus pasteurianus* (100%) |
|  | OTU20 | 0.006 | 0.009 | 0.023 | 0.432 | *Prevotella paludivivens* (91.04%) |
|  | OTU21 | 0.006 | 0.009 | 0.023 | 0.112 | *Prevotella oris* (91.51%) |
|  | OTU24 | 0.007 | 0.009 | 0.013 | 0.208 | *Prevotella oris* (93.40%) |
|  | OTU25 | 0.012 | 0.012 | 0.004 | 0.907 | *Bacteroides stercoris* (95.05%) |
|  | OTU27 | 0.011 | 0.006 | 0.008 | 0.444 | *Faecalibacterium prausnitzii* (99.75%) |
|  | OTU29 | 0.015 | 0.005 | 0.009 | 0.482 | *Prevotella stercorea* (92.22%) |
|  | OTU30 | 0.006 | 0.008 | 0.006 | 0.706 | *Sporobacter termitidis* (90.66%) |
|  | OTU31 | 0.010 | 0.008 | 0.006 | 0.483 | *Hungateiclostridium thermocellum* (90.66%) |
|  | OTU32 | 0.005 | 0.003 | 0.002 | 0.553 | *Bacteroides coprocola* (91.98%) |
|  | OTU33 | 0.013 | 0.002 | 0.003 | 0.206 | *Prevotella timonensis* (92.45%) |

1 OTUs with relative abundances higher than 1% within total bacteria were sorted and showed in the table.

^a, b, c^ Mean values within a line with different superscript letters differ significantly (*p* < 0.05).

Table S4 Relative abundance of microbial OTUs (percentage) in the colon mucosa of piglets under different feeding frequencies (n = 8) ^1^.

| OTU Name | M1 | M3 | M5 | *P*-value | Annotation |
| --- | --- | --- | --- | --- | --- |
| OTU1 | 0.188 | 0.232 | 0.196 | 0.064 | *Streptococcus pasteurianus* (100%) |
| OTU2 | 0.026 | 0.044 | 0.068 | 0.369 | *Lactobacillus johnsonii* (100%) |
| OTU3 | 0.037 | 0.034 | 0.047 | 0.344 | *Lactobacillus reuteri* (99.77%) |
| OTU4 | 0.021 | 0.029 | 0.040 | 0.553 | *Frisingicoccus caecimuris* (98.51%) |
| OTU5 | 0.015 | 0.022 | 0.009 | 0.527 | *Clostridium saudiense* (98.27%) |
| OTU6 | 0.016 | 0.021 | 0.017 | 0.505 | *Hungateiclostridium thermocellum* (90.66%) |
| OTU7 | 0.012 | 0.004 | 0.011 | 0.785 | *Bacteroides coprocola* (91.98%) |
| OTU8 | 0.017 | 0.009 | 0.006 | 0.066 | *Prevotella oralis* (92.92%) |
| OTU9 | 0.012 | 0.017 | 0.008 | 0.498 | *Terrisporobacter petrolearius* (99.26%) |
| OTU10 | 0.017 | 0.004 | 0.002 | 0.675 | *Alloprevotella rava* (92.29%) |
| OTU11 | 0.007 ^b^ | 0.013 ^a^ | 0.011 ^ab^ | 0.037 | *Streptococcus pasteurianus* (99.77%) |
| OTU12 | 0.007 | 0.005 | 0.008 | 0.633 | *Faecalibacterium prausnitzii* (99.75%) |
| OTU13 | 0.009 | 0.011 | 0.010 | 0.174 | *Streptococcus pasteurianus* (98.60%) |

1 OTUs with relative abundances higher than 1% within total bacteria were sorted and showed in the table.

^a, b^ Mean values within a line with different superscript letters differ significantly (*p* < 0.05).

Table S5. Summary of DEGs associated with metabolism in ileum and colon mucosa.

| Tissues | Group | Genes ^1^ | Major Metabolic Types |
| --- | --- | --- | --- |
| Jejunum  mucosa | M3 VS M1 | ***ADRA2C, NDUFAF2, PRKAA2, ADRB2****, CHRM4,* ***CHST13, GAL3ST4, GALR1, GALR3, NPPC, ST8SIA2, St8sia4,*** *PFKFB3,* *PPP1R3C,* ***SDHAF3,*** *SLC5A4,* *TRIB3,* ***AIPL1,*** ***CEP290,*** *EPHA2,* ***GAL3ST4,*** ***CASD1*** | Carbohydrate metabolism |
|  |  | ***ACSM4****, DHCR24, FA2H,* ***FCER1A****,* ***GAL3ST4, PIK3C2G, PRKAA2,*** *SREBF1****, ST8SIA2, St8sia4,*** *TRIB3, VDR, ACOX1,* ***ADRB2****, CLPS,* ***CRABP1, CYP26A1, CYP2C42, HPGDS, PLCL1,*** *RBP2****,*** ***TMEM55A, ATG4C****,* ***BRINP3, CD80, LTF, SFR1,*** *TNFAIP3,* ***ZFP36L2,*** *SYT7,* ***ABCB11, ATP8B4, GALR1,*** *NOS2, OSBP2,* ***HGF*** | Lipid metabolism |
|  |  | *GPT2,* ***ADRA2C, CD80, CDC7, DCLK3,*** *EPHA2,* ***FCER1A, FKBP7, HGF, INSRR,*** *NOS2,* ***PLCL1, PRDM5, PRKAA2,*** *SPRED2,* ***TAF9B, WNK3, YEATS4, ATG4C,*** *ASS1,* ***DCT, RRAGD, SLC38A11*** | Amino acid metabolism |
|  | M5 VS M1 | *IFNG, ACACB, ADCY2,* ***ADRB3, GAL3ST4, GUCA1B,*** *NME3, OAS2,* ***ST8SIA2,*** ***AIPL1,*** ***CMAH,*** *COL2A1, CXCL11, AQP5* | Carbohydrate metabolism |
|  |  | *ABCA1, APOA1, APOC3, ACACB, FDPS,* ***GAL3ST4,*** *IFNG,* ***SPTSSB, ST8SIA2, HPN,*** *PLCE1,* ***PLCH2, GAL3ST4, CASP9,*** *IL12A,* *PAQR9,* *TNFAIP3,* ***ZFP36L2,*** *ANO6, CXCL11, SLC10A6, FDPS, OAS2* | Lipid metabolism |
|  |  | *GPT2, CLIC6, FOXE1, GDAP1L1,* ***IYD, HAL,*** *MTR,* *APOA1,* ***CRLF1,*** *FKBP6, IFNG, IL12A, LIF,* ***MAPK9,*** *NFATC2IP, PRDM12,* ***TTBK1,*** *TTBK2, SLC6A18* | Amino acid metabolism |
|  | M5 VS M3 | *DPY19L1, EOGT, MAN1A2, PGM2L1, ST3GAL4, St8sia4, TUSC3,* ***SLC2A6,*** *RHOQ, CASD1, CHI3L2, GALR1,* ***PIGP,*** *CEP290, KL* | Carbohydrate metabolism |
|  |  | *ACSL4, ITGAV, ENPP7,* ***SMPD1,*** *ATG4C, GALR1, St8sia4,* ***PIGP,*** *ABCA1,* *FDPS, PTGDS, SCD5,* *ATP8B4, NYAP2, SELP, PLCL1,* ***USF1,*** *CD80, SFR1, STAP1, CYP26A1, LACTB* | Lipid metabolism |
|  |  | *DCT,* ***IYD,*** *DUOX2, GDAP1L1, GSTA4,* ***SARDH,*** *RRAGD, ZEB1,* ***SLC7A4,*** *SLC38A11, TRPC4, CD80, DPY19L1, INSRR, JADE1, KIT,* ***P3H2,*** *PAQR3, PLCLI,* ***PRDM14,*** *PRMT3, PTPRC, RIFI, SETDB2, SOCS5, ST3GAL4, STAP1, TTBK2, TTLL7, TUSC3, ATG4C, RRAGD,* | Amino acid metabolism |
| Colon  mucosa | M3 VS M1 | *MTMR2, ADAMTS12, EDEM3, LYVE1,* ***AHCYL2,*** ***CHST13, CMAH,*** *HRH4,* ***IHH,*** *ITIH4, RAMP2,* ***SDHD,*** *SHAS2,* ***SYVN1,*** *UGGT2,* ***VIP,*** *LOC100737622,* ***PAX6,*** ***PRKAA2,*** *GNPTG, LBP,* ***APOA2,*** ***SLC5A11*** | Carbohydrate metabolism |
|  |  | *APOA1,* ***APOA2, APOA4,*** *ANXA1, bmp4, CCL21, CXCL2,* ***CYP24A1,*** *KCNJ8, LBP,* ***MSTN,*** *NR0B2, PPBP, fp, ATP10B, PLA2G12A, PLA2G2C, MTMR2,* ***EPHX2,*** *BMP5,* ***CYP26A1,*** *HSD17B7,* ***PRKAA2,*** *PTGDS, RDH12,* | Lipid metabolism |
|  |  | ***SLC1A1, ICMT,*** *ABI2,* *APOA1,* ***APOA2, HRG,*** *FLT4,* ***IFN-omega-4,*** ***IL2, MYLK4,*** *PECAM1, PLK2,* ***PRKAA2,*** ***SENP5, SYVN1,*** *UGGT2,* ***AHCYL2,*** *CLIC6, VNN2,* ***TRPC4,*** *ARG1* | Amino acid metabolism |
|  | M5 VS M1 | ***FFAR3, PRKAA2, PTCH1, ADCY1, ADRB2, CHST13, FOXL1,*** *CMPK2,* ***St8sia4,*** *GUCA1A, OAS2,* ***AHCYL2, CMAH,*** *KL* | Carbohydrate metabolism |
|  |  | ***ADRB2, CAV1,*** *CMPK2,* ***FFAR3, JUNB,*** *KCNK4,* ***NCOA3,*** *NR0B2,* ***PTCH1,*** *PTGER1,* ***SMO, ACSM4, AGMO, ATM, CDS2, INSIG1, NPC1L1, PRKAA2, SPTLC3, St8sia4, FAM126A, CIDEA, PLIN4, SYT7, SERPINA6,*** *OAS2,* ***ZDHHC19*** | Lipid metabolism |
|  |  | ***HPD, GPT2, MYOCD, NCOA3, YEATS2, FOLR1, ICMT, RNF152, CAV1, CHD5, DCX, IFN-ALPHA-14, IFN-omega-6, INSR, PRKAA2, SENP5, ZDHHC19, AHCYL2,*** *CGA* | Amino acid metabolism |
|  | M5 VS M3 | ***MLXIPL, ADAL, ADCY1, ALG14, AQP1, CAP2,*** *CMPK2,* ***EDEM3, FOXL1,*** *GUCA1A, NME8,* ***NPR1,*** *OAS2,* ***RAMP2, SHAS2, UGGT2, ADAMTS12, ATP1A2, PCSK6, UGGT2,*** *SLC2A2,* *GSTO1,* ***MLXIPL,*** *OAS2,* ***PTCH1*** | Carbohydrate metabolism |
|  |  | ***ANXA1, AQP1, ATP1A2, CAV1,*** *CMPK2,* ***CXCL2,*** *CYP24A1,* ***EPHA3, MRC1, NCOA3, PTCH1,*** *SETX,* ***SLC11A1, SMO, AGT, CDS2,*** *CYP11A1,* ***FDFT1, FGFR4, HSD17B7, INSIG1, MLXIPL, PTGIS, SLC27A2, SPTLC3, sqle, STAR, ABCB11, PLIN4, PRELID2, SYT7, TNFAIP8L3, ABHD1, ALG14, DGKE, FAM126A, FDFT1,*** *LIPE,* ***SERPINA6,*** *OAS2* | Lipid metabolism |
|  |  | ***HPD, ABI2, ACE, AGT,*** *ARNT,* ***CAV1, CHD5, DCX, DMPK, EFEMP1, EPHA3, FGF10, FGFR4, IFN-ALPHA-14,*** *CGA, GSTO1,* ***MTHFD2L, VNN2, RNF152, COL3A1, MYOCD, NCOA3,*** *TRPC4* | Amino acid metabolism |

^1^ Genes formatted in bold were up-regulated, while the other genes were down-regulated. DEGs, differentially expressed genes.
